# Coupling of a viral K^+^-channel with a glutamate-binding-domain highlights the modular design of ionotropic glutamate-receptors

**DOI:** 10.1038/s42003-019-0320-y

**Published:** 2019-02-22

**Authors:** Michael Schönrock, Gerhard Thiel, Bodo Laube

**Affiliations:** 1 Department of Biology, Neurophysiology and Neurosensory Systems, Technische Universität Darmstadt, 64289 Darmstadt, Germany; 2 Department of Biology, Plant Membrane Biophysics, Technische Universität Darmstadt, 64289 Darmstadt, Germany

## Abstract

Ionotropic glutamate receptors (iGluRs) mediate excitatory neuronal signaling in the mammalian CNS. These receptors are critically involved in diverse physiological processes; including learning and memory formation, as well as neuronal damage associated with neurological diseases. Based on partial sequence and structural similarities, these complex cation-permeable iGluRs are thought to descend from simple bacterial proteins emerging from a fusion of a substrate binding protein (SBP) and an inverted potassium (K^+^)-channel. Here, we fuse the pore module of the viral K^+^-channel Kcv_ATCV-1_ to the isolated glutamate-binding domain of the mammalian iGluR subunit GluA1 which is structural homolog to SBPs. The resulting chimera (GluATCV*) is functional and displays the ligand recognition characteristics of GluA1 and the K^+^-selectivity of Kcv_ATCV-1_. These results are consistent with a conserved activation mechanism between a glutamate-binding domain and the pore-module of a K^+^-channel and support the expected phylogenetic link between the two protein families.

## Abstract (web summary)

Michael Schönrock et al. created a functional chimeric channel composed of the pore domain of the potassium channel, KcvATCV-1, and the glutamate binding domain of the mammalian ionotropic glutamate receptor, GluA1. This chimera recognized glutamate as a ligand while displaying the potassium selectivity of KcvATCV-1; highlighting the modular design of ionotropic glutamate receptors.

## Introduction

**Fig. 1:**
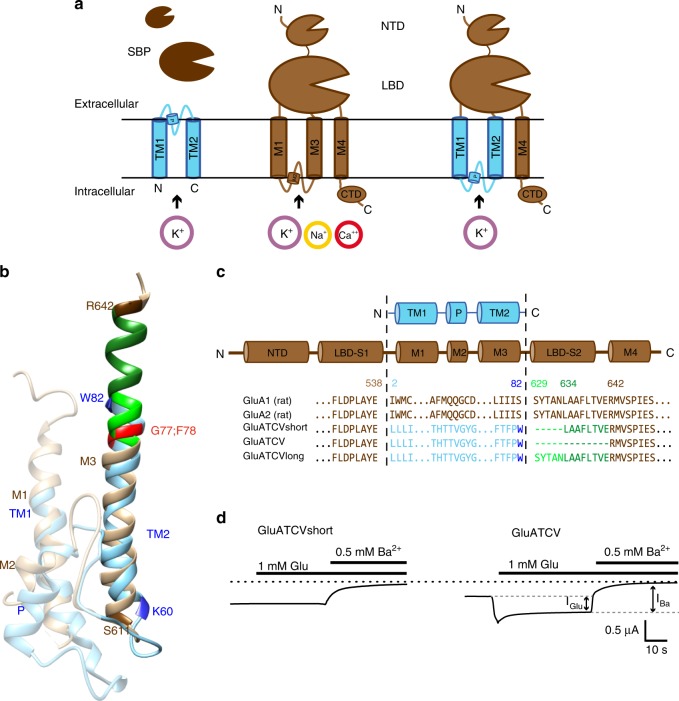
Design of Kcv_ATCV-1_/GluA1chimeras. **a** The cartoon depicting the topology of a subunit of the Kcv_ATCV-1_ (blue), GluA1 (brown), and the chimera GluATCV harboring the membrane-spanning domain of the Kcv_ATCV-1_. Amino-terminal domain (NTD), ligand-binding domain (LBD), C- terminal domain (CTD), pore helix (P), transmembrane domains (TM respectively M in case of GluA1), substrate-binding-protein (SBP, dark brown). Permeant cations are indicated. **b** Structural overlay TM of Kcv_ATCV-1_ and GluA2. TM1 and pore helix as well as M1 and M2 of GluA2 are transparent. TM of Kcv_ATCV-1_ (TM2) and GluA2 (M3) are in full color. Helices were superimposed by aligning main-chain atoms of TM2 segments from Kcv_ATCV-1_ model (see Methods) on crystal structure of M3 domain of GluA2 subunit (Protein Database entry 3KG2^20^) (side view). Backbones and residues of subunits are illustrated in ribbon representations (blue: Kcv_ATCV-1_; brown: GluA2) within iGluRs conserved SYTANLAAF region (green) and position of G77 and F78 of Kcv_ATCV-1_ (red). **c** Design of GluATCV constructs. Partial sequence alignment of GluA1, GluA2, and different GluATCV constructs. Illustration of amino acid cutting sites of different GluA1/Kcv_ATCV-1_ chimeras (GluATCV). Chimeras harboring different lengths of the SYTANLAAF motif  (green) with corresponding residue numbering of mature subunits (Kcv_ATCV-1_ blue; GluA1 brown) are indicated. GluATCVlong (linker length + 13 aa); GluATCVshort (linker length + 8 aa), and GluATCV (no linker). Residues found at cutting sites and within the SYTANLAAF motif are highlighted. Secondary structure elements found are illustrated above the sequence. **d** Functional characterization of chimeric GluATCV constructs. Representative whole-cell currents of glutamate (Glu) responses and Ba^2+^ inhibition of GluA1/Kcv_ATCV-1_ chimeras upon heterologous expression in *Xenopus* oocytes recorded at −70 mV membrane potential. Oocytes expressing GluATCVshort and GluATCV were superfused with the indicated concentration of glutamate in the absence and presence of 500 µM Ba^2+^. Current traces illustrating inhibitory effects of Ba^2+^ in both chimera. Note that K^+^-specific blocker Ba^2+^ inhibits both glutamate-induced currents elicited from GluATCV and leak current in GluATCVshort expressing oocytes. Black dotted line indicates Ba^2+^ insensitive leakage current. Bars show timepoint and duration of the application. Gray dotted line and arrows indicate glutamate induced current (*I*_Glu_) and barium blockable current (*I*_Ba_)

Conversion of a chemical to an electrical signal is a hallmark of neurons in the central nervous system (CNS). This process is mediated by different families of ligand-gated ion-channels, membrane-spanning proteins that open upon recognition of a specific neurotransmitter. In particular, gating of cation-permeable receptors by the amino acid glutamate (iGluRs) mediating excitatory neuronal signaling in the mammalian CNS is crucially involved in both, learning and memory formation and pathological mechanisms leading to excitotoxic neuronal damage in diverse neurological diseases^1^. Because mutations within subunits are associated with a number of diseases of the nervous system, these receptors are of interest as a major class of targets for novel drugs. The mammalian iGluR family includes three major subtypes represented by several subunits: AMPA (α-amino-3-hydroxyl-5-methyl-4-isoxazolepropionic acid; GluA1-4), kainate (GluK1-5) and NMDA (N-methyl-D-aspartate; GluN1, GluN2A-D, and GluN3A-B) receptors. Crystallographic and Cryo-EM studies in recent years have provided detailed structural information of these membrane-spanning iGluRs (overview in refs. ^2,3^). They are tetrameric complexes composed of homologous subunits. Each subunit has a conserved modular design and is composed of an extracellular N-terminus (NTD), an extracellular ligand-binding domain (LBD), an intracellular C-terminus (CTD), three transmembrane domains (M1, M3, and M4), and one pore loop (M2)^3^. The latter inserts into the membrane from the intracellular side and forms together with the M3 segment the channel pore^4^. The modular architecture of iGluRs suggests that they have descended from a common ancestral prokaryotic receptor. Notably the complex domain architecture of all eukaryotic iGluRs is partially conserved in a simple bacterial iGluR from *Synechocystis* (called GluR0)^5^, indicating an origin of all eukaryotic iGluRs before the prokaryote–eukaryote dichotomy occurred. However, although a structural overall similarity between the LBD and the transmembrane domains composed of the segments M1–M3 has been proposed^5,6^, the bacterial GluR0 greatly differs from its eukaryotic counterparts and lacks the NTD, M4, and CTD. Interestingly, the LBD and channel pore domain of iGluRs are structurally related to other bacterial proteins, i.e., the substrate binding proteins (SBP) and potassium (K^+^)-channels, respectively^7–9^. For both bacterial protein families, crystallization has yielded detailed structures with related function and highlighted the idea that SBPs and K^+^-channels are functional homologous to the LBD and the M1–M3 segment of iGluRs, respectively. Therefore an evolutionary link between these bacterial protein families and the GluR0 was proposed which might arise by the insertion of an inverted K^+^-channel pore between the SBP domains leading to the formation of a modular prototype of potassium-specific channels (i.e., GluR0)^5^ (see for illustration Fig. 1a). Prokaryotic SBPs facilitate chemotaxis and substrate uptake of a large variety of small molecules and ions by binding their ligands with high specificity and affinity. Interestingly, SBPs share also a common ligand binding mechanism with the LBD of iGluRs consisting of two-lobed domains (S1 and S2) connected by a hinge forming a clamshell-like structure^7^. Ligand binding takes place at the interface between the two domains, inducing a domain closure. This conformational change functions as the key element in the transition of ligand recognition and ion channel gating in iGluRs^10,11^. K^+^-channels are selective for potassium ions and comprise a large family of ion channels. All K^+^-channels share the same core topology and tetrameric structure and differ only in the presence or absence of additional transmembrane helices and of additional non-membrane domains. The basic channel-forming core is composed of two transmembrane helices (TM1 and TM2; called S5 and S6 in voltage-dependent potassium (Kv) channels) with low sequence similarity between the different K^+^-channels. The TM1 and TM2 domains are separated by a pore loop (P-loop), which inserts into the membrane from the extracellular side and contains a conserved “TXXTVGYG” signature sequence of all K^+^-channel selectivity filters^12,13^. However, although K^+^-channels and the pore-forming core domain of iGluRs share a common architecture, the K^+^-channel selectivity filter is not conserved and the pore-forming core domain of the K^+^-channelshows an inverted orientation in the membrane. Nevertheless, the apparent sequence and structural similarities of the different domains led to the hypothesis that the GluR0 is a modular composition of an inverted bacterial K^+^-channel and a SBP and represents the precursor of mammalian iGluRs^8^. However, a functional compatibility between the pore structure of a K^+^-channel and the LBD of an iGluR could yet not been demonstrated, although several studies tried without success to fuse the LBD of iGluRs with diverse K^+^-channels. Based on these negative results the hypothesis of a compatible architecture of iGluR and K^+^-channel pores was rejected^14,15^. Here, we revisit the possibility of fusing the membrane-spanning domain of the small viral Kcv_ATCV-1_ potassium channel to the glutamate-binding LBD of the AMPA iGluR subunit (GluA1) for creating a functional glutamate-gated potassium channel. It has been shown that small viral K^+^-channels are because of their structural simplicity, functional robustness and the absence of any coevolution with cellular proteins most suitable as building blocks in synthetic channels where they maintain their conductive properties in the presence of attached regulatory domains^16–18^. With this approach, we can show that the fusion of the LBD of GluA1 to the minimal K^+^-channel pore of Kcv_ATCV-1_ generates a truly glutamate-gated K^+^-channel. Collectively this provides experimental support for the hypothesis of a phylogenetic link between iGluRs and K^+^-channels. It also implies a conserved activation mechanism of the pore region of iGluRs and ancestral viral K^+^-channels, which is gated by mechanical coupling to the LBD.

## Results

### Design of glutamate-gated GluA1/Kcv_ATCV-1_ chimeras

To investigate a putative functional compatibility of the pore structure of K^+^-channels and mammalian iGluRs, we substituted the channel forming transmembrane domain region of the AMPA GluA1 by a minimal K^+^-channel of viruses named Kcv_ATCV-1_ for K^+^-channel from chlorella virus (see for illustration Fig. 1a). These viral channel proteins have the structural and functional hallmarks of pro- and eukaryotic K^+^-channels^19^, but are composed of only 82 amino acids and likely constitute the minimal unit of a functional K^+^-channel pore module. A structural overlay of a homology model of the Kcv_ATCV-1_ (see Methods) on the crystal structure of the transmembrane domains of the GluA2 subunit (Protein Database entry 3KG2^20^); in combination with published sequence alignments (see refs. ^21–23^) revealed that the TM2 helix of the Kcv_ATCV-1_ is about eight amino acids shorter than the M3 and TM2 domain of canonical iGluR and K^+^-channels, respectively. Remarkably, the last amino acids of the C-terminal helix of TM2 in Kcv_ATCV-1_ overlap with the N-terminal sequence of the highly conserved SYTANLAAF motif in iGluR linking the M3 to the LBD (Fig. 1b^24^). Inspired by this homology we generated an initial AMPA GluA1 receptor chimera (GluATCV_short_; Fig. 1c) by inserting the Kcv_ATCV-1_ pore at position 538 and 634 of the GluA1. In this construct the TM2 helix of the viral protein was extended by the rest of the C-terminal part of the SYTANLAAF motif to match the total M3 length of iGluRs. To avoid glutamate-mediated channel desensitization, we used a construct containing the desensitization attenuated mutant L479Y in the LBD of the GluA1^25,26^ in all following experiments.

When we analyzed GluATCV_short_ by two-electrode voltage clamping in *Xenopus laevis* oocytes, we could not elicit any currents in response to 1 mM glutamate (Glu). Remarkably, compared to control oocytes, we observed a large leakage current (*I*_leak_) in the range of 0.9 ± 0.3 µA in the absence of the agonist which could be efficiently blocked by the specific K^+^-channel blocker Ba^2+^ (93.5 ± 1.6%; *n* = 5; Fig. 1d; Table 1)^27^, but not with the specific competitive glutamate antagonist CNQX (6-cyano-7-nitroquinoxaline-2,3-dione). From these data we reasoned that the pore domain of the Kcv_ATCV-1_ converted the GluATCV_short_ construct into a K^+^-channel-like behavior, which, however, could not be gated by external Glu. Since the M3-LBD linker is a crucial element in iGluR gating^10^, we generated two additional constructs with variable lengths of the TM2 helix (Fig. 1c). Strikingly, although the new construct with a 13 amino acid deletion including the SYTANLAAF motif (GluATCV; Fig. 1c) showed again a high leakage current (0.6 ± 0.2 µA; Table 1), it responded in a robust manner to Glu. Application of the ligand elicited currents with 0.4 ± 0.3 µA in amplitude (Fig. 1d; *n* = 10). In contrast, the construct containing the whole SYTANLAAF motif (GluATCV_long_; Fig. 1c) behaved similar to the initial GluATCV_short_ construct and remained insensitive to Glu application. Both, the initial leakage and the Glu-evoked currents of the GluATCV could be blocked by Ba^2+^ (Fig. 1d). Quantitative analysis of the Ba^2+^-sensitive Glu-induced response and the resting leakage current (*I*_Glu_/*I*_Ba_) revealed that 36.4 ± 4.6% (*n* = 5) of the total Ba^2+^-sensitive currents in the GluATCV construct could be specifically induced by Glu-application (Table 1). The results of these experiments indicate that the substitution of the pore-forming transmembrane domains of the GluA1 by a minimal viral K^+^-channel resulted in a Glu-gated K^+^-channel with a relatively high spontaneous activity in the resting state.

**Table 1:** Leakage currents and fractional Ba^2+^-sensitivity of control and GluATCV_short_, GluATCV and GluATCV* expressing oocytes

	Uninjected	GluATCVshort	GluATCV	GluATCV*
**Initial leakage current [µA]**	0.1 ± 0.06	0.9 ± 0.3	0.6 ± 0.2	0.2 ± 0.1
**Ba** ^**2+**^ **-block of the leakage current [%]**	<10%	93.5 ± 1.6	84.4 ± 2.3	32.9 ± 10.8***
**Glutamate-induced fraction of the Ba** ^**2+**^ **-block [%]**	n.d.	n.d.	36.4 ± 4.6	68.2 ± 5.2**

Note that the fractional contribution of the Ba^2+^-sensitive leakage current to the overall leakage and of the Glu-induced current to the total Ba^2+^-sensitive current are highly significantly different between GluATCV and GluATCV* expressing oocytes (**, *p* = 0.0017; unpaired two-side *t*-test; ***, *p* < 0.0001; unpaired two-side *t*-test; *n* = 5). *n.d.* not detectable

### Increase of glutamate efficacy

**Fig. 2:**
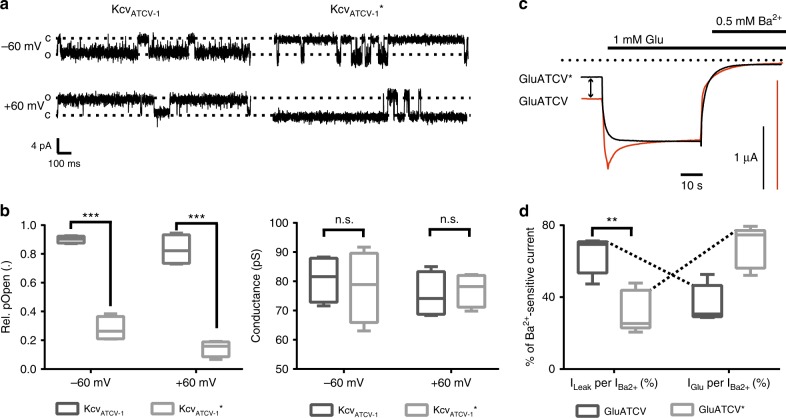
Increase of Glu-gating efficiency in GluATCV by TM2 point mutations. **a** Single channel recordings of the Kcv_ATCV-1_ and Kcv_ATCv-1_*. Currents of Kcv_ATCV-1_ and Kcv_ATCV-1_* (mutated at aa positions (G77S, F78L)) were recorded at a membrane potential of +60 and −60 mV upon reconstitution in planar lipid bilayer (see Methods). Note the difference of the open probability in the characteristic single channel fluctuations of the two K^+^ channels. **b** Analysis of the open probability and single channel conductance of Kcv_ATCV-1_ and Kcv_ATCV-1_*. Plot of the open probabilities (Po) and single channel conductance (pS) of the wt and mutant Kcv_ATCV-1_ channel by calculating the time of occupancy of the open state (O) and the closed state (C) from 4 independent 1 min recordings at +60 and −60 mV (*p* < 0.001; unpaired two-side *t*-test; *n* = 4). **c** Overlay of representative recordings of glutamate (Glu) responses and Ba^2+^ inhibition at GluATCV (red) and GluATCV* (black). Arrow illustrates the differences in the ratio of the inhibition of the glutamate-induced currents and the resting leakage by the K^+^-specific blocker Ba^2+^ in GluATCV and GluATCV* expressing oocytes. Dotted line indicates the Ba^2+^ insensitive leakage current. **d** Fractional contribution of the Ba^2+^-sensitive leakage- and of Glu-induced currents in GluATCV constructs. Percentage of the Glu-induced currents of the total Ba^2+^-sensitive current in GluATCV* is highly significant increased compared to the GluATCV (68.2 ± 5.2% vs. 36.4 ± 4.6%, respectively; *p* = 0.0017; unpaired two-side *t*-test; 95% confidence interval = −47.74 to −15.94; *n* = 5)

It is known that Kcv_ATCV-1_ has a high intrinsic open probability (Po)^19^, suggesting that the high leakage current of the GluATCV construct might be due to a high intrinsic spontaneous activity of the Kcv_ATCV-1_ pore domain. To test this hypothesis, we exploited a recent finding of the related Kcv_NTS_ channel, where two substitutions had been shown to greatly reduce Po^28^. We therefore analyzed the open probability of the wt (wildtype) Kcv_ATCV-1_ and the corresponding double-mutant Kcv_ATCV_* by functional reconstitution in planar lipid bilayers. Consistent with the data of the related Kcv_NTS_, substitution of G77 to S and F78 to L caused also in the Kcv_ATCV-1_ a significant reduction in the Po from 0.83 ± 0.11 to 0.14 ± 0.01 in the Kcv_ATCV_* mutant at −60 mV (Fig. 2a, b; *p* < 0.001; unpaired two-side *t*-test; 95% confidence interval = −0.8497 to −0.569; *n* = 4) without altering single channel conductance (79 ± 6.1 and 81 ± 4.0 pS for the GluATCV and GluATCV*, respectively; Fig. 2b). In the next step we tested whether these mutations alter the ratio *I*_Glu_/*I*_Ba_ in the GluATCV construct. The data in Fig. 2c show that the double mutant G614S/F615L in the GluATCV* (* for low intrinsic spontaneous activity) generates indeed the expected effect. GluATCV* generated currents with a drastic reduced leakage component (*I*_leak_ = 0.2 µA ± 0.1; *n* = 5) and a concomitant increase in the amplitude of the Glu-inducible current (Fig. 2c, d; *I*_Glu_ = 0.7 ± 0.2 µA; *n* = 5). Again, both, the glutamate-induced and the leakage currents could be efficiently blocked by Ba^2+^ (Fig. 2c) underscoring that both currents are generated by the GluATCV* channel. Obviously, by reducing the spontaneous activity of the pore domain in the GluATCV* construct, indicated by the 2-fold decrease in the Ba^2+^-sensitive leakage currents (Fig. 2d), the contribution of the Glu-induced current to the total current increased by a factor of 2 (GluATCV* 68.2 ± 5.2% vs. GluATCV 36.4 ± 4.6%; Fig. 2d; Table 1; *p* = 0.0017; unpaired two-side *t*-test; 95% confidence interval = −47.74 to −15.94; *n* = 5). Remarkably, although the fractional contribution of the Ba^2+^-sensitive leakage and Glu-mediated currents were inversely affected in the GluATCV and the GluATCV* constructs, the maximal Ba^2+^-sensitive currents were not different (GluATCV 1.0 ± 0.3 µA vs. GluATCV* 0.8 ± 0.2 µA). The results of these experiments suggest that the overall expression and activity of the two constructs were similar and that a high intrinsic spontaneous activity of the channel pore hampers the efficiency of the Glu-mediated channel opening in our GluATCV constructs.

### Pharmacological characterization of the GluATCV*

**Fig. 3:**
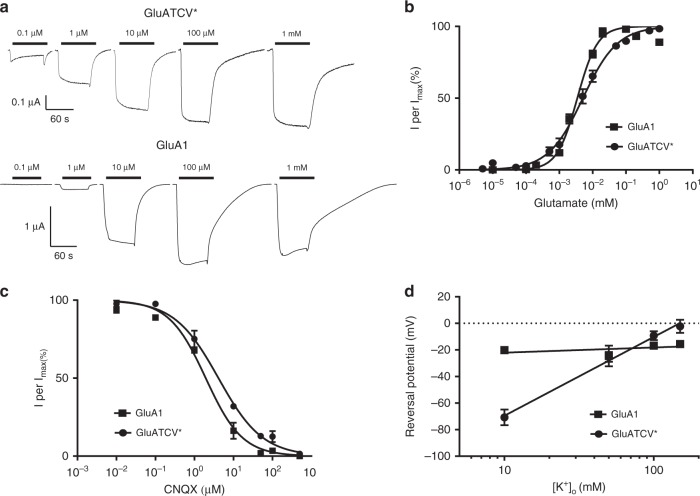
Functional characterization of the GluATCV chimera. **a** Glu responses of GluA1 and GluATCV* upon expression in *Xenopus* oocytes. Traces of Glu responses of GluA1 and GluATCV* with different concentrations of glutamate. The bars show the duration of the application of the corresponding concentration. **b** Glutamate dose–response curves recorded from GluA1 (squares) or GluATCV* (circles) expressing oocytes. Both show a similar EC_50_ value of 5.8 ± 1.2 and 4.0 ± 0.4 µM for GluATCV* and GluA1, respectively. **c** CNQX-inhibition curve of GluA1 and GluATCV* at the EC_50_ value of glutamate. The CNQX IC_50_ is 4.4 ± 0.9 and 2.0 ± 0.1 µM for GluATCV* and GluA1, respectively. **d** Plot of the reversal voltages of the GluA1 and GluATCV* receptors against the extracellular K^+^ concentration. Reversal voltages were estimated by current–voltage (*I*–*V*) recordings in different ringer solutions containing 10, 50, 100, and 150 mM K^+^. The proportional shift of the reversal voltage as a function of the concentration of K^+^ in the extracellular medium with a slope of 59.3 ± 4.9 for the GluATCV* and 3.9 ± 4.7 for GluA1 confirms a high selectivity for K^+^ over Na^+^ in the GluATCV* channel

For a first pharmacological characterization of the GluATCV*, we determined glutamate dose–response curves and calculated the respective EC_50_ (half maximal effective concentration) values for the parental GluA1 and GluATCV*. Remarkably, neither the resulting Glu-induced current traces nor the EC_50_ values of the GluATCV* were distinguishable from those of the GluA1 receptor (Fig. 3a, b). Both showed desensitization attenuated Glu-currents with similar EC_50_ values (5.8 ± 1.2 and 4.0 ± 0.4 µM for the GluATCV* and GluA1, respectively; *p* = 0.2; *n* = 8). Similarly, inhibition curves with the competitive AMPA antagonist CNQX revealed an IC_50_ (half maximal inhibitory concentration) value comparable to wt GluA1 (4.4 ± 0.9 and 2.2 ± 0.1 µM for the GluATCV* and GluA1, respectively; *p* = 0.1; unpaired two-side *t*-test; 95% confidence interval −5.045 to 0.6263; *n* = 5; Fig. 3c). These data show that the insertion of a Kcv_ATCV-1_* channel pore did neither affect (i) the amplitude of the current responses nor (ii) the apparent agonist and antagonist-affinity. In summary, the results of these experiments show that the GluATCV* pharmacology is similar to the native GluA1 channel.

In further experiments, we examined the ion selectivity of GluATCV*. Since the GluA1 channel exhibits no selectivity for K^+^ over Na^+^^29^, we tested whether insertion of the Kcv_ATCV-1_* channel converts the unspecific GluA1 monovalent cation channel into a specific K^+^-selective channel by ion substitution. Analysis of Glu-induced current/voltage (*I*/*V*)-curves in bath solutions in which K^+^ was replaced by Na^+^ showed that the GluATCV* caused, different to GluA1, a shift of the reversal voltage as a function of the extracellular K^+^ concentration: the shift in reversal voltage of GluATCV* can be fitted with a Nernst equation providing a mean shift of 59.3 ± 4.9 for a 10-fold increase in [K^+^]_o_. In the same experiments GluA1 only responds with a shift of 3.9 ± 4.7 to a 10-fold change in [K^+^]_o_ (Fig. 3d). This shift of the reversal voltage confirms a high selectivity for K^+^ over Na^+^ in the GluATCV* channel. The P(Na^+^)/P(K^+^) ratio can be calculated by the Goldman–Hodgkin–Katz equation under bi-ionic conditions and is with 0.04 ± 0.01 similar to that of Kcv_ATCV-1_^27^, but significantly different from that of the nonselective GluA1 channel (P(Na^+^)/P(K^+^) of 0. 87 ± 0.02; *p* < 0.05; see also refs. ^30,31^). Collectively our results show that the GluATCV* chimera functions as a K^+^-selective, glutamate-gated channel. It combines both, the characteristics of ligand recognition of the GluA1 iGluR and the selectivity of the Kcv_ATCV-1_.

### Design of a minimal glutamate-gated potassium channel

**Fig. 4:**
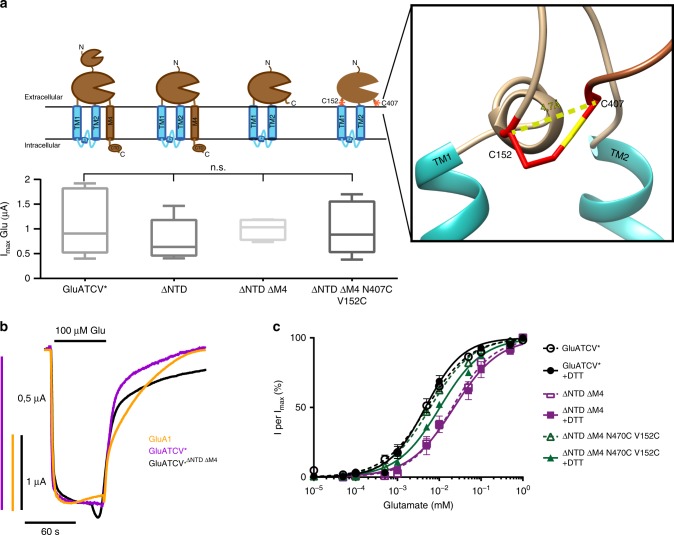
Design of minimal Glu-gated viral potassium channels. **a** Schematic drawings and functional expression of the deletion and mutant constructs used. Cartoons depicting the NTD- and the M4 truncated versions and the cysteines mutated (indicated by red stars); Inset: Point mutations in the LBD (N407C) and TM1 segment (V152C) thought to form a disulfide-bridge are highlighted in red based on our homology model against 3KG2. Numbers correspond to amino acid positions in the mature protein. *I*_max_ currents of each mutant, with and without DTT in the case of the cysteine double mutant, are shown (*p* = 0.8; one-way ANOVA with turkey correction; *n* = 4–10). **b** Overlay of representative whole cell current traces of Glu-gated GluATCV*, GluATCV*^ΔNTD ΔM4^, and GluA1. The respective traces in response to application of 100 µM glutamate (Glu) are shown in magenta, black, and orange, respectively. Note that the overall shape of the glutamate-induced whole cell currents is similar between the constructs. **c** Glu dose–response curves of GluATCV* (black circles), the deletion mutant GluATCV*^ΔNTD ΔM4^ (magenta squares), and the double mutant GluATCV*^ΔNTD ΔM4 V152/N402C^ (green triangles) without DTT (open symbols) and in the presence of 5 mM DTT (closed symbols). EC_50_ GluATCV* 5.8 ± 1.1 µM (−DTT) and 5.1 ± 1.1 µM (+DTT); GluATCV*^ΔNTD ΔM4^ 20.8 ± 2.6 µM (−DTT) and 22.6 ± 2.9 µM (+DTT); GluATCV*^ΔNTD ΔM4 V152/N402C^ 5.9 ± 0.4 µM (−DTT) and 10.5 ± 0.7 µM (+ DTT). Note that, in contrast to GluATCV* and GluATCV*^ΔNTD ΔM4^, only the GluATCV*^ΔNTD ΔM4 V152/N402C^ shows a significant changed EC_50_ value in the presence of DTT (green closed triangles) indicative of an intrasubunit disulfide link between LBD and TM1

To investigate the minimal structural requirements for a functional Glu-gated Kcv channel we successively truncated GluA1 domains. First, we examined whether an N-terminally truncated GluATCV* construct that lacks amino acids 19–395 of the mature subunit (GluATCV*^ΔNTD^, Fig. 4a) forms functional receptors in *X. laevis* oocytes. Figure 4a shows that receptors composed of the NTD-deleted GluATCV*^ΔNTD^ subunit still displayed robust agonist responses. In the presence of saturating glutamate concentrations, the *I*_max_ values were similar to those of the full-length GluATCV* receptor (Fig. 4a; *I*_max_ = 1.0 ± 0.26 µA; *p* = 0.8; one-way ANOVA with turkey correction; *n* = 5), indicating that the N-terminal domain is neither essential for GluATCV assembly nor for function. Next, a C-terminal truncated GluATCV*^ΔNTD^ subunit was generated by deleting residues downstream of V784 including transmembrane domain M4 and the whole C-terminal domain (GluATCV*^ΔNTD ΔM4^; Fig. 4a). Although 55% of the initial GluATCV construct is deleted in the GluATCV*^ΔNTD ΔM4^, we observed desensitization-attenuated currents with similar *I*_max_ values after applying Glu at saturating concentrations (Fig. 4a, b). Since these currents are comparable to those generated by the GluA1 and the full-length GluATCV* receptors (Fig. 4b), we reasoned that neither the NTD nor the C-terminal domain (including M4) of the GluA1 in the GluATCV* construct are required for receptor assembly, membrane insertion, and functional expression. This prompted us to determine the apparent glutamate affinities of the truncated constructs. The analysis shows that the Glu-affinities of GluATCV* and GluATCV*^ΔNTD ΔM4^ receptors were 3-fold different with EC_50_ values of 5.8 ± 1.2 vs. 20.8 ± 2.6 µM, respectively (*p* < 0.001; unpaired two-side *t*-test; 95% confidence interval −0.02056 to −0.009614; *n* = 4; Fig. 4c). The affinity in the GluATCV*^ΔNTD^ receptor on the other hand was similar to that of the full-length channel (5.8 ± 1.0 µM). Based on current models of iGluR activation, we assume from these data that anchoring of the LBD to the M4 may be essential for high-affinity Glu-binding to GluATCV receptors. To test the importance of LBD–TM interactions for receptor stabilization, we identified in the GluA2 (PDB entry 3KG2) based GluATCV homology model (Methods) two amino acids in the N-terminal part of TM1 (V152) and at the very C-terminus of the LBD (N407) (Fig. 4a inset). The side chains of these amino acids are directed into the center of the subunit towards an intra-subunit cavity. We measured that the Cα atoms are separated by less than 5 Å. This implies that a disulfide bond between the LBD and the TMs could be formed for stabilizing LBD–TM interactions. To test this hypothesis the two residues were substituted by cysteine. The resulting GluATCV*^ΔNTD ΔM4 V152C/N407C^ construct and the GluATCV* and GluATCV*^ΔNTD ΔM4^ receptors were expressed in oocytes and the Glu-current response recorded in the absence and presence of dithiothreitol (DTT), which reduces disulfide bonds between cysteine residues^32^. The resulting Glu-dose–response curves in the absence of DTT were indistinguishable from those of the M4 containing GluATCV* receptors (GluATCV* and GluATCV*^ΔNTD^). Remarkably, after DTT treatment, only the EC_50_ value of the GluATCV*^ΔNTD ΔM4 V152C/N407C^ receptor showed a significant 2-fold decrease in apparent Glu-affinity (from 5.9 ± 0.4 to 10.5 ± 0.7 µM; *p* = 0.001, unpaired two-side *t*-test; 95% confidence interval −0.006532 to −0.002572; *n* = 6) whereas the EC_50_ values of the GluATCV* and the GluATCV*^ΔNTD ΔM4^ receptors were not affected by DTT (Fig. 4c). At the same time, the maximal inducible currents of all constructs tested were not significantly changed by the addition of DTT. These data are consistent with the idea that the pair of cysteines, which were introduced in GluATCV*^ΔNTD ΔM4 V152C/N407C^ receptors, is able to form a disulfide bond. Breaking of this bond by DTT resulted in a decrease in apparent Glu-affinity. The results of these measurements suggest that stabilizing of LBD–TM interactions via a linker (exemplified at position 152 and 407) contributes to ligand affinity of GluATCV receptors, in an otherwise complete functional state of the receptor.

## Discussion

Our data demonstrate that a naive coupling of the LBD of the mammalian GluA1 subunit from the ionotropic glutamate receptor family (iGluR) with the viral potassium channel Kcv_ATCV-1_ generates a K^+^-selective, glutamate-gated receptor channel. This functional compatibility of the LBD of an iGluR with the minimal pore domain of a K^+^-channel underscores a modular architecture with a conserved activation mechanism within the two families and highlight their phylogenetic link. A functional coupling of the two orthogonal domains also provides a tool to understand the most basic mechanical interactions between ligand recognition and channel gating irrespective of co-evolutionary constrains. Ultimately it may foster the design of new K^+^-selective biosensors for specific analyte recognition.

For many years it has been suggested that the pore modules of K^+^-channels and iGluRs share a similar architecture and that they might have a common evolutionary ancestor^8,9^. This idea of a direct phylogenetic link has been supported by similarities in amino-acid sequences and homologies in structural details of the respective pore domains^13,33–36^. However, an experimental affirmation of this hypothesis by demonstrating a functional convertibility of an iGluR and a K^+^-channel was not provided so far. In contrast, data from several studies argue against a compatible architecture of iGluR and K^+^-pores^14,15^. Here, we can show that a chimeric construct between the LBD of the mammalian GluA1 and the viral potassium channel Kcv_ATCV-1_ functions efficiently as a K^+^-selective, glutamate-gated ion channel with the specific characteristics of ligand recognition of the iGluR and the ion selectivity of the K^+^-channel. This result strongly supports the idea of a modular design of iGluRs in which the pore originated from an ancestral K^+^-channel. Furthermore, the results shed some interesting light on a common molecular mechanism for the activation of P-loop channels in general. In particular, our initial rationale to use a minimal viral K^+^-channel for linking an iGluR LBD was, that the helical segment of the inner TM2 in Kcv_ATCV-1_ is too short to interfere with the endogenous iGluR channel gate (SYTANLAAF-motif gate; overview in refs. ^10,35,37^). Therefore, we assumed that including the SYTANLAAF motif of the iGluR in our constructs might render the viral K^+^ channel sensitive to LBD-mediated gating. We find however that this motif is not essential for Glu-mediated K^+^-channel gating; it can be substituted by an endogenous, so far not anticipated K^+^-viral gate.

Glutamate-gated channel opening is thought to be mediated via an iris-like conformational change in the TMs^38^ by pulling the M3 pore-forming helices away from the central pore axis upon glutamate-binding^1,2,4,20^. This proposed mechanism is comparable with K^+^-channel gating^39,40^. This simple mechanical model, which is supported by molecular dynamics simulations and combined functional and computational studies^41,42^, predicts that the closed-to-open free energy of the channel should be related to the tension within the LBD-M3 linker; the channel open probability should be determined by the LBD-M3 linker length. However, based on recent cryo-EM studies in the GluA2^38^ this view of a simple action of the linkers as a rigid mechanical lever has been challenged. Here, based on the 2-fold symmetrical arrangement of the LBDs, the S2-M3 linkers connecting the LBDs to the pseudo-4-fold symmetrical channel form two conformational distinct diagonal pairs and contribute geometrically and energetically different to channel opening^38^. The differential contribution of the linker pairs results in an inherent asymmetric state of the M3 helices, which is similar to a recently described pairwise asymmetric dimer of dimers state proposed by a kink in the inner TM2 domain in viral potassium channels^28^. Thus, although the gating hinges in the TM2 of potassium channels and in the M3 of iGluRs seem to be different and structural unique, we think that the 2-fold changes in the M3 of iGluRs mediated by LBD dimers and the pairwise asymmetric state in the TM2 during viral K^+^-channel gating are converted to channel opening; this mechanism seems to be conserved in tetrameric P-loop ion channels. This may sufficiently explain the functionality of our minimal construct by a phylogenetically conserved, pairwise adopted asymmetric channel configuration during channel opening below the principal rotational symmetry of the pore.

The present data provide implications for our understanding of iGluR assembly. For several years it was thought that the tetrameric assembly of iGluRs is mediated by inter-subunit interfaces including the NTD, LBD and the TMD segments (overview in ref. ^3^). This simple view has been questioned by experimental data. By using deletion constructs it has been shown that the NTDs do not play the important role in iGluR assembly^43,44^ which was previously anticipated. The importance of the transmembrane domains in oligomerization remains still controversial. As an additional player interactions of the TMs with the M4 segment have been implicated as important for subunit assembly^43–48^. The present data show that constructs, in which M4 was deleted, are still able to assemble into functional glutamate-gated channels. This argues against a critical role of the M4 for proper receptor assembly. This finding is consistent with data obtained for the NMDAR^45,46,49^, where deletion of the M4 domain did not affect receptor assembly and surface expression. Together, these findings are also in line with the functional subunit structure of a bacterial iGluR (i.e., GluR0 from *Synechocystis*), which contains only two TM segments (M1 and M3) and no NTD.

It is worth mentioning that a construct, in which the M4 domain was deleted, displayed a decreased apparent glutamate affinity. This indicates a role of the S2-M4 linker or of the M4 domain in transducing agonist efficacy to channel opening. As already mentioned, changes in the LBD layer are transmitted to ion channel opening by means of the LBD–TM linkers. Although the most important changes seem to occur in the M3-S2 linkers, it has been shown in the GluA2 structure that changes are also observed in S2-M4 linkers^38^. This is consistent with either a contribution of the S2-M4 linkers and/or the M4 segment in gating kinetics or a simple stabilization of the LBD via a rigid TM anchor. We find that breaking and forming of a disulfide bond between two cysteines of the C-Terminus of S2 and the TMs affected the apparent glutamate affinity, indicating that anchoring of the LBDs may affect M3-S2 linker tension.

Finally, our results are consistent with a modular architecture of iGluRs and a phylogenetic link between these proteins and K^+^-channels. Chiu and colleagues^50^ proposed that the core function of iGluRs occurred before animals and plants separated from a common albeit unknown prokaryotic ancestor. The present data support the view that a key stage in the evolution of iGluRs was presumably the insertion of an inverted potassium channel pore domain between the two parts (S1 and S2) of a bacterial SBP. Despite a low sequence identity, SBPs are structural homologous to the LBD of iGluRs and share a common ligand binding mechanism. Thus, it can be assumed that the fusion of the two proteins may have occurred in prokaryotes. This is reflected in the K^+^-selective GluR0 channel lacking the NTD and M4 domains. Indeed, homology modeling and simulation studies of GluR0 supports a degree of common fold and functional similarity between the pore-forming region in GluR0 and the prokaryotic K^+^-channel KcsA^51^. In this study, we report the functional characterization of GluATCV, where a eukaryotic iGluR LBD was functionally linked with success to a K^+^-channel pore. Interestingly this was achieved by using a viral potassium channel, e.g., a channel which is from a structural point of view very simple and evolutionary ancient^52^. Collectively these data support the idea of an ancient phylogenetic link between iGluRs and K^+^-channels. The present data furthermore underscore that a primitive channel with poor control on gating can acquire sophisticated ligand-mediated regulation via a naive coupling with an orthogonal LBD. This proof of concept finding makes it likely that ligand gated channels occurred as a result of a singular evolutionary step. Following this line of thought we also predict that synthetic GluATCV channels can now offer an excellent experimental system for understanding structure/function correlates in complex glutamate receptors. In a learning-by-building approach the synthetic channels can now be engineered in such a way that they exhibit the functional behavior of complex glutamate receptors. In this way it will be possible to understand in an unbiased manner the basic structural interactions between the sensing and the pore unit, which are crucial for the function of complex glutamate receptors.

## Methods

### Materials

CNQX (6-cyano-7-nitroquinoxaline-2,3-dione) was purchased from Tocris (Biotrend, Cologne, Germany), all further chemicals were purchased from Sigma (Taufkirchen, Germany). Restriction enzymes, Phusion polymerase and T4 ligase were purchased from Thermo Fisher (Waltham, USA).

### DNA constructs, oocyte expression and TEVC

*Rattus norvegicus* GluA1 glutamate receptor carrying the mutation L479Y (Genebank ID EDM04494.1) provided by R. Sprengel (MPI for medical research, Heidelberg) and the K^+^ channel from chlorella virus Kcv_ATCV-1_ (GeneID 5470584) were subcloned into the expression Vector pEXP5-NT/TOPO by using the pEXP5-CT/TOPO ® TA Expression Kit of Invitrogen. The GluATCV constructs were generated by replacing the nucleotide sequence encoding amino acids 538–629 (GluATCVlong), 634 (GluATCV short) and 642 (GluATCV) of the mature GluA1 by the Kcv_ATCV-1_ sequence with the use of XhoI and NheI. The GluATCV^ΔNTD^ and GluATCV^ΔM4^ constructs were generated by excising the nucleotide sequence encoding amino acids 19–394 and upstream of 784 of the mature GluA1 by deletion PCR, respectively. All constructs were confirmed by DNA sequencing (Seqlab, Göttingen, Germany).

cRNAs were synthesized by using the AmpliCap-Max™ T7 High Yield Message Maker Kit of Cellscript (Madison, Wi, USA) with the plasmid linearized by AatII. *X. laevis* oocytes were used for two electrode voltage-clamp (TEVC) electrophysiology as previously described^53^. Oozytes were surgically obtained from female *X. laevis* after anesthesia with 0.1% Tricaine in water under the approval of the Technical University of Darmstadt (Agreement V54-19c20/15 DA8/Anz. 20). After the harvest, oocytes were incubated for 1 h in 0.8 mg collagenase in frog ringer [96 mM NaCl, 2 mM KCl, 1 mM CaCl_2_,1 mM MgCl_2_, and 5 mM HEPES (pH 7.4 with NaOH)]^53^. After the treatment and a washing step with Ca^2+^ free ringer [96 mM NaCl, 2 mM KCl,1 mM MgCl_2_, and 5 mM HEPES (pH 7.4 with NaOH)] oocytes of stage V and VI were selected and defolliculated by pipetting with a burned glass-Pasteur-pipette. For electrophysiological analysis, oocytes were injected with 50 ng in a volume of 50 nl of the respective construct. After injection the oocytes were incubated in ND-96 solution (96 mM NaCl, 2 mM KCl, 1 mM CaCl_2_, 1 mM MgCl_2_, 5 mM HEPES, pH 7.4 with NaOH) at 18 °C for 3–5 days until the electrophysiological measurements. The TEVC recordings were performed at room temperature with an Axoclamp 900A amplifier, digitized with a Digidata 1550A at 5 kHz after low-pass filtering at 200 Hz and recorded with Clampex 10.7 (Molecular Devices, San Jose, USA). For recording the microelectrodes were filled with 3 M KCl (resistance 0.8–2.8 MΩ in external solution) and the oocytes were clamped at −70 mV. The external solution was a modified ringer solution containing 100 mM KCl; 10 mM HEPES; 1.8 mM CaCl_2_; 1 mM MgCl_2_ (pH 7.4 with KOH) alone or containing agonist. L-glutamate, CNQX, and Ba^2+^ were applied to the oocytes in external solution at the given concentrations. Dose–response curves of CNQX were determined in the presence of 5 µM glutamate (corresponding to the EC_50_ value) and normalized to the current in the absence of CNQX. Whole-cell current–voltage relationships of saturating glutamate-induced currents were recorded in ramps from −140 mV to 140 mV with 14 mV/100 ms in 2 s in solutions with different concentrations of potassium (substituted by sodium) and corrected by the current values obtained in the absence of glutamate. For treatments with DTT, oocytes were superfused with 2 mM DTT for 100 s before applying glutamate in the presence of 2 mM DTT as described by Lynagh et al.^53^.

### Protein production and Bilayer measurements

The proteins of Kcv_ATCV-1_ and Kcv_ATCV-1_* were produced in an in vitro transcription reaction with the MembraneMax™ HN Protein Expression Kit in presence of nanolipoproteins in accordance with manufacturer’s instruction^54^. The reaction was purified by His-tags fused to the nanolipoproteins over a HisPur NI-NTA spin column (Thermo Scientific, Waltham, USA) after manufacturer protocol and eluted with 250 mM imidazole. For bilayer experiments a dilution of 1:5000 in 250 mM imidazole was used.

Bilayer experiments were performed in a vertical planar lipid bilayer chamber^54^ and channel activity was measured with an eOne Amplifier from Elements s.r.l., (Cesena, Italy) under symmetric conditions with solution containing 100 mM potassium chloride with 10 mM HEPES pH 7.4. Planar lipid bilayers were formed over a hole of ca. 100 µm in a 20 µm Teflon foil, which was pretreated with 1% hexadecane solution in n-hexane. Bilayers were made from 1,2-diphytanosyl-sn-glycero-3-phosphocholine (DPhPC, from Avanti Polar Lipids, Alabaster, Alabama) diluted at 15 mg/ml in n-pentane with the folding technique^55^. The phospholipid solution was therefore added as monolayer on the measure solution and after evaporation of the solvent a bilayer was folded by raising the solutions in the chambers. After formation of a bilayer, the electrical activity of the empty membrane was monitored at a voltage of ±100 mV to exclude contaminations or lipid pores. Only when the bilayer was stable and electrically silent a small amount (1 µl) of protein (Kcv_ATCV-1_ or Kcv_ATCV-1_*) in nanodiscs diluted 1:5000 in 250 mM imidazole was added to the trans chamber near the membrane with a Hamilton syringe. After insertion of an active channel the voltage protocol was applied again and the resulting currents were recorded with Ag/AgCl electrodes. The data were digitized at 5 kHz after lowpass filtering at 2.5 kHz.

### Analysis

Current responses to glutamate were plotted against glutamate concentration and fit with non-linear regression with variable slope in Prism version 7.00 (GraphPad Software Inc., La Jolla, USA) as described^56^. The proportion of glutamate sensitive current to complete barium sensitive current was calculated by *I*_Glu_/*I*_Ba_. Permeability ratio was calculated by the shift of the reversal potential with the Goldman–Hodgkin–Katz equation under bi-ionic conditions $\frac{{P_{\mathrm{Na}^ + }}}{{P_{\mathrm{K}^ + }}} = {\mathrm{exp}}\left( {\Delta E_{\mathrm{rev}} \ast \frac{F}{{R \ast T}}} \right)$^27^. The amplitude was measured with KielPatch and also the open probability in bilayer measurements was analyzed by the build in Hinkley-jump-detector of KielPatch (http://www.zbm.uni-kiel.de/aghansen/software.html). Values given represent means ± SEM. Statistical significance was determined at the *p* < 0.05 (*), *p* < 0.01 (**), and *p* < 0.001 (***) levels using a Student’s two-tailed, unpaired two-side *t*-test or ANOVA (in case of more groups) and a Tukey post-hoc analysis (Prism 7.00). All data are shown as boxplots with a center line representing the median; box limits are the upper and lower quartiles; whiskers representing min and max.

### Structure model

The homology model of Kcv_ATCV-1_ was made with Swiss-model against the KirBac1.1^57^, the homology model of GluATCV* ΔM4 was built against the GluA2 structure 3KG2 also with swiss-model and the structure overlays was built with UCSF Chimera^58^ and the MatchMaker plugin with default values. Distance of C152/C407 mutants was measured in this homology model of GluATCV* ΔM4 by the chimera distance tool between Cα atoms.

### Reporting summary

Further information on experimental design is available in the Nature Research Reporting Summary linked to this article.

## Supplementary information

Reporting Summary

Supplementary Data 1

Description of Additional Supplementary Files

## Data Availability

The authors declare that the data generated during this study are available in the manuscript and the figures. Source data underlying the graphs presented in the figures are available in Supplementary Data 1. All cDNA constructs are available from the corresponding author based on reasonable request.
