# Supplementary material for: Coupling of a viral K+-channel with a glutamate-binding-domain highlights the modular design of ionotropic glutamate-receptors
Source: Commun Biol. 2019 Feb 22;2:75. doi: 10.1038/s42003-019-0320-y (PMC6385376; doi:10.1038/s42003-019-0320-y)
Supplement: Supplementary file 3 — Description of Additional Supplementary Files [file 42003_2019_320_MOESM3_ESM.docx]

Description Supplementary Data1

The Supplementary Data 1 includes the source data for all graphs of the figures in this paper.
